# The Oncolytic Caprine Herpesvirus 1 (CpHV-1) Induces Apoptosis and Synergizes with Cisplatin in Mesothelioma Cell Lines: A New Potential Virotherapy Approach

**DOI:** 10.3390/v13122458

**Published:** 2021-12-08

**Authors:** Iris Maria Forte, Paola Indovina, Serena Montagnaro, Aurora Costa, Carmelina Antonella Iannuzzi, Francesca Capone, Rosa Camerlingo, Anna Maria Malfitano, Francesca Pentimalli, Gianmarco Ferrara, Massimiliamo Quintiliani, Giuseppe Portella, Antonio Giordano, Roberto Ciarcia

**Affiliations:** 1 Cell Biology and Biotherapy Unit, Istituto Nazionale Tumori, IRCCS, Fondazione G. Pascale, 80131 Naples, Italy; c.iannuzzi@istitutotumori.na.it (C.A.I.); r.camerlingo@istitutotumori.na.it (R.C.); f.pentimalli@istitutotumori.na.it (F.P.); 2 Institute for High Performance Computing and Networking, National Research Council of Italy (ICAR-CNR), 80131 Naples, Italy; paola.indovina@icar.cnr.it; 3 Sbarro Institute for Cancer Research and Molecular Medicine, Center for Biotechnology, College of Science and Technology, Temple University, Philadelphia, PA 19122, USA; 4 Department of Veterinary Medicine and Animal Productions, University of Naples “Federico II”, 80137 Naples, Italy; semontag@unina.it (S.M.); gianmarco.ferrara@unina.it (G.F.); r.ciarcia@unina.it (R.C.); 5 Department of Medical Biotechnologies, University of Siena, 53100 Siena, Italy; aurora.costa@student.unisi.it; 6 Experimental Pharmacology Unit—Laboratories of Naples and Mercogliano (AV), Istituto Nazionale per lo Studio e la Cura deiTumori “Fondazione G. Pascale”, 80131 Naples, Italy; f.capone@istitutotumori.na.it; 7 Dipartimento Scienze Mediche Traslazionali, Università di Napoli “Federico II”, 80131 Naples, Italy; annamaria.malfitano@unina.it (A.M.M.); portella@unina.it (G.P.); 8 Department of Life, Health and Environmental Sciences, University of L’Aquila, 67100 L’Aquila, Italy; mquintiliani@unite.it

**Keywords:** malignant mesothelioma (MM), oncolytic virus (OV), caprine herpesvirus 1 (CpHV-1), cisplatin, synergism, apoptosis

## Abstract

Malignant mesothelioma (MM) is an aggressive asbestos-related cancer, against which no curative modalities exist. Oncolytic virotherapy is a promising therapeutic approach, for which MM is an ideal candidate; indeed, the pleural location provides direct access for the intra-tumoral injection of oncolytic viruses (OVs). Some non-human OVs offer advantages over human OVs, including the non-pathogenicity in humans and the absence of pre-existing immunity. We previously showed that caprine herpesvirus 1 (CpHV-1), a non-pathogenic virus for humans, can kill different human cancer cell lines. Here, we assessed CpHV-1 effects on MM (NCI-H28, MSTO, NCI-H2052) and non-tumor mesothelial (MET-5A) cells. We found that CpHV-1 reduced cell viability and clonogenic potential in all MM cell lines without affecting non-tumor cells, in which, indeed, we did not detect intracellular viral DNA after treatment. In particular, CpHV-1 induced MM cell apoptosis and accumulation in G0/G1 or S cell cycle phases. Moreover, CpHV-1 strongly synergized with cisplatin, the drug currently used in MM chemotherapy, and this agent combination did not affect normal mesothelial cells. Although further studies are required to elucidate the mechanisms underlying the selective CpHV-1 action on MM cells, our data suggest that the CpHV-1-cisplatin combination could be a feasible strategy against MM.

## 1. Introduction

Malignant mesothelioma (MM) is a very aggressive tumor developing from the mesothelium covering the body cavities. The most common MM type affects the pleura surrounding the lungs. MMs are classified into three main histologic subtypes: epithelioid, sarcomatoid, and biphasic, which are characterized, respectively, by epithelial cells, spindle-shaped cells, or both cell types [1,2], and with sarcomatoid tumors determining the poorest outcome [2,3].

MM is mainly associated with asbestos exposure [4]. Although the use of asbestos has been banned in several countries, MM incidence is increasing due to both the long latency time between exposure and tumor occurrence and the persistence of environmental exposure [4]. Moreover, asbestos is still used in developing countries and the use of other asbestos-like fibers that can cause MM, such as erionite, is not strictly regulated [3].

Despite featuring a predominant etiology linked to asbestos exposure, MM is highly heterogeneous at the molecular level, which is a key hurdle in developing effective therapies [1,5,6,7,8]. Indeed, although several treatment strategies have been explored and many promising therapeutic targets have been identified over the years [7,9,10,11,12], standard systemic treatment still consists of platinum-based chemotherapy plus pemetrexed, combined, only in carefully selected patients, with surgery and radiation, which has demonstrated limited effects [12,13,14]. Thus, the overall survival (OS) rates remain approximately 9–12 months from diagnosis [4,15]. Therefore, there is an urgent need to identify new effective therapeutic strategies for this tumor.

Oncolytic virotherapy is a promising therapeutic approach that uses engineered viruses to treat several malignancies and has recently been applied successfully in the clinical setting [16,17,18,19]. Oncolytic viruses (OVs) selectively replicate in cancer cells and kill them with a multimodal mechanism of action, without affecting normal cells [20]. MM represents a good candidate for this strategy owing to the pleural accessibility it offers for the intra-tumoral injection of the OV [21]. We recently demonstrated that the oncolytic adenovirus *dl922-947* expressed antitumor effects in both MM cell lines and mouse xenografts and synergized with cisplatin [22], the drug currently used in MM chemotherapy, and also with AZD1775, a drug targeting the DNA damage response induced by the OV [23].

Besides adenovirus, herpesvirus or reovirus have also demonstrated oncolytic properties [24,25,26]. In particular, talimogenelaherparepvec (T-VEC), an attenuated oncolytic herpes simplex virus type 1 (oHSV-1), armed with granulocyte-macrophage colony-stimulating factor (GM-CSF), has been approved as the first OV treatment for unresectable stage IIIB to IV melanoma [27]. Indeed, oHSV-1 is one of the most studied viruses for cancer therapy, owing to its ability to infect a wide range of host cells, express transgene products with excellent efficiency, deliver multiple transgenes, and grow at high titers [24,25]. However, oHSV-1 infection can induce side effects, such as fatigue, nausea, influenza-like illness, vomiting, and headache [18,28]. Moreover, owing to its intrinsic neurotropism, oHSV-1 could have neurotoxic effects on normal brain cells [29,30]. Therefore, oHSV-1 is generally attenuated by engineering, with a consequent reduction in pathogenicity, but also in oncolytic ability [28].

Among the different types of OVs now available, some non-human wild-type OVs present advantages over human OVs, including the inability to replicate in normal human cells while having a natural tropism for human cancer cells and the absence of pre-existing immunity [31]. For instance, the Newcastle disease virus (NDV) is an avian paramyxovirus, which poses no threat to human health. NDV has a lytic replication cycle in human tumor cells associated with antitumor immune responses. In particular, tumor cells are susceptible to NDV infection, owing to defects in cellular interferon (IFN) signaling and apoptotic pathways [32]. Moreover, the rat protoparvovirus H-1PV is non-pathogenic in humans and replicates preferentially in cancer cells, in which it shows oncolytic and oncosuppressive effects. The virus activates several cell death pathways and triggers anticancer immune responses [33].

Among these non-human wild-type OVs, caprine herpesvirus 1 (CpHV-1) is a pathogen of goats, closely related to bovine alphaherpesvirus 1 (BoHV-1), which is non-pathogenic for humans [34]. We previously demonstrated that CpHV-1 is able to replicate in different human cancer cell lines and kill them by apoptosis or autophagy [35].

Here, we tested the effects of CpHV-1 on a panel of MM cell lines, both alone and in combination with cisplatin, demonstrating not only the ability of this OV to replicate in MM cells and kill them, without affecting normal mesothelial cells, but also that the CpHV-1 and cisplatin combination could be a feasible strategy against MM.

## 2. Materials and Methods

### 2.1. Cell Lines and Culture Conditions

The NCI-H28, MSTO-211H, and NCI-H2052 mesothelioma cell lines, the MET-5A mesothelial cells transfected with an SV40 ori-construct containing the SV40 early region, the Rous sarcoma virus long terminal repeat, and the Madin Darby bovine kidney (MDBK) cells were purchased from American Type Culture Collection (ATCC; Manassas, VA, USA). The MM cells were grown in RPMI-1640 supplemented with 10% fetal bovine serum (FBS), 1% penicillin-streptomycin, and 1% glutamine. The MET-5A cells were grown in Medium 199 with 10%FBS, 0.5% penicillin-streptomycin, 1% glutamine, and 3.3 nM epidermal growth factor, 400 nM hydrocortisone, and 870 nM insulin. The MDBK were maintained in high-glucose Dulbecco’s modified Eagle’s medium supplemented with 10% fetal bovine serum (FBS), 1%penicillin-streptomycin, and 1% glutamine. All the cell culture reagents were obtained from Sigma-Aldrich (St. Louis, MO, USA). The cells were maintained at 37 °C in a humidified atmosphere containing 5% CO_2_ and were routinely tested with a PlasmoTest^TM^ Mycoplasma Detection kit (Cat. no. rep-pt1; Invivogen, San Diego, CA, USA) for the presence of mycoplasma.

### 2.2. Virus Production

The reference Swiss strain E/CH was used [35,36]. The virus was multiplied on the MDBK cell line. The cell extracts, obtained by three cycles of freezing and thawing, were pooled, collected, and stored in aliquots at −80 °C. Before use, a viral solution was partially purified by centrifugation at 3000 rpm for 20 min to eliminate cell debris and then pooled and stored in aliquots at −80 °C [37]. Infectivity titers were expressed as median tissue culture infectious doses (TCID50)/mL [38].

Viral production in the mesothelial and MM cell lines was assessed by harvesting the cells and supernatants at 24 h, 48 h, 72 h, 96 h, and 120 h upon infection with 5 MOI of CpHV-1. Viral production was then assessed in the MDBK cells and expressed as median tissue culture infectious doses (TCID50/mL). The cytopathic effect (CPE) was scored and calculated using the Reed and Muench method [38].

### 2.3. Cell Infection with CpHV-1, MTS, and Clonogenic Assay

The NCI-H28, MSTO-211H, NCI-H2052, and MET-5A cells were seeded in triplicates in 96 well plates at a density of 800 cells/well (MSTO-211H) or 1200 cells/well (NCI-H28, NCI-H2052, MET-5A) and allowed to adhere for 24 h. The cells were then infected with CpHV-1 at doses ranging from 0.1 to 5 MOI for 24, 48, 72, 96, and 120 h. After treatment, the cells’ viability was evaluated by MTS assay (cat. no. G3582; CellTiter 96^®^ AQueous One Solution Cell Proliferation Assay, Promega, Milan, Italy), following the manufacturer’s instructions.

For the clonogenic assay, 200 cells were seeded in each well of 6 well plates and, 24 h after seeding, they were treated with CpHV-1 for 72 h at 5 MOI. After 10 days, colonies were fixed with methanol and stained at room temperature for 30 min with crystal violet (Cat. no. HT90132; Sigma-Aldrich). At this cell density, the MET-5A did not form quantifiable clones.

### 2.4. Viral DNA Extraction and Quantification by Real-Time PCR

The MSTO-211H and MET-5A cells were seeded in 60 mm dishes (5 × 10^5^ cells/well) and 24 h later infected with viruses at 5 MOI and 10 MOI. The cells and supernatants were collected 48 h and 72 h p.i. (either separately or together). The cell pellets were disrupted by three freeze-thaw cycles to release the virus; they were then centrifuged at 1000× *g* for 5 min and the supernatants were collected. Viral DNA was extracted through Viral RNA isolation (Cat. No. 740956.50; Macherey–Nagel, available online: https://www.mn-net.com/nucleospin-rna-virus-mini-kit-for-viral-rna-from-cell-free-fluids-740956.50 accessed on 22 September 2021) and amplified by real-time PCR, using the following primers: forward, 5′-AAACAGGAATTAACTATACTAATATATTTA-3′, and reverse, 5′-AAATTTGACCATTTGGATAAACT-3′, in both the supernatants and the cellular pellets.

### 2.5. Cytofluorimetric Analysis of Cell Cycle Profile and Cell Death

For the cell cycle analysis, all the cell lines were infected with CpHV-1 at 5 MOI and 72 h p.i. were collected, washed with PBS, and then fixed in 70% ice-cold ethanol. The cells were then incubated at 37 °C for 1 h with 50 μg/mL propidium iodide (PI; cat. no. P4170; Sigma-Aldrich) and 20 μg/mL RNase (Cat. no. 9001-99-4; Sigma-Aldrich) and then analyzed with BD FACSAria™ III and BD FACSDiva Software 8.0 (BD Biosciences, San Jose, CA, USA).

For apoptosis detection, the cells were stained with Annexin V-FITC and PI (cat. no. 130-092-052; Annexin V-FITC kit; Miltenyi Biotec Inc., Bologna, Italy) according to the manufacturer’s instructions and analyzed by FACS (BD FACSCalibur, BD Biosciences).

### 2.6. Protein Extraction and Western Blot Analysis

For total protein extraction, the cells were lysed on ice for 30 min in a lysis buffer containing 1 mM EDTA, 150 mM NaCl, 1%NP-40, 50 mM TRIS-HCL pH 7.5, and 10 mg/mL each of aprotinin, leupeptin, and pepstatin. Equal amounts of proteins (50 μg) per sample were subjected to SDS-PAGE. Western blots were performed with antibodies against PARP (cat. no. 556494, mouse monoclonal; BD PharMingen, Franklin Lakes, NJ, USA), Cleaved PARP (Asp214) (cat. No. #5625, rabbit monoclonal, Cell Signaling Technologies, Danvers, MA, USA), Caspase-3 (cat. no. #9662, rabbit polyclonal; Cell Signaling, Danvers, MA, USA), and GAPDH (cat. no. sc-25778, rabbit polyclonal; Santa-Cruz, Dallas, TX, USA). The signals were detected through ECL (cat. no. 34580; Amersham Biosciences, Amersham, UK). The intensity of the bands was quantified by densitometric analysis using ImageJ software, available online: https://imagej.nih.gov/ij/download.html (accessed on 22 September 2021).

### 2.7. Drug Combination Studies

We treated the MM cells for 72 h with CpHV-1 and cisplatin, both alone and in combination at various concentrations in a constant ratio, and assessed cell viability through MTS assay. Synergism, additivity, or antagonism were determined through an isobologram analysis using the CompuSyn software 1.0 (CompuSyn, Inc., Paramus, NJ, USA, available online: https://compusyn.software.informer.com/1.0/ accessed on 22 September 2021). The CI values were also calculated according to the Chou–Talalay equation, using the CompuSyn software. CI < 1 indicates synergism, CI = 1 additivity, and CI > 1 antagonism. The *r* value represents the linear correlation coefficient of the median—effect plot, which indicates the conformity of the data to the mass–action law.

### 2.8. Statistical Analysis

The statistical analyses were performed using GraphPad Prism Software, version 5.01 for Windows available online https://www.graphpad.com/scientific-software/prism/ (accessed on 22 September 2021). Statistically significant differences between multiple matched groups were evaluated by one-way repeated measures ANOVA with a Bonferroni post-test. Values of *p* < 0.05 were considered statistically significant.

## 3. Results

### 3.1. CpHV-1 Reduces MM Cell Viability and Clonogenic Potential

**Figure 1:**
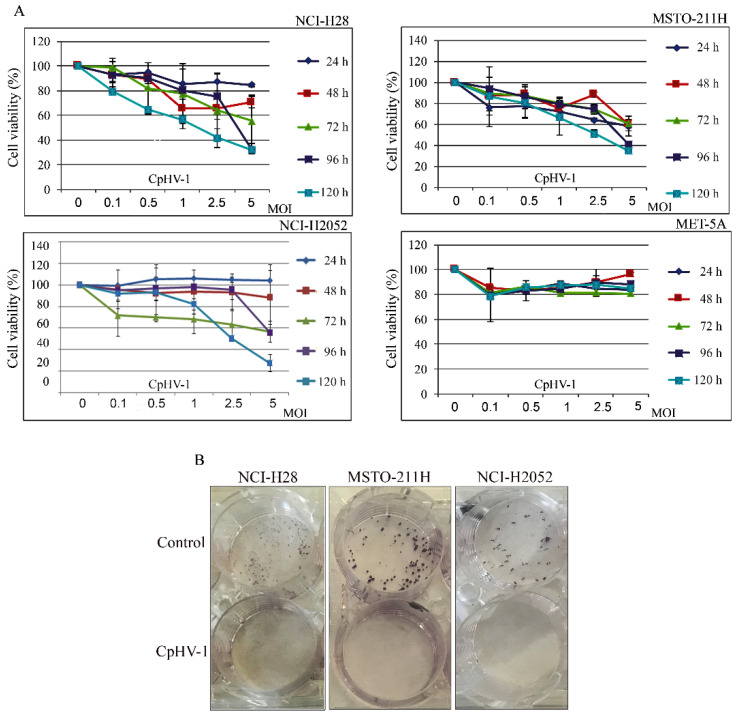
Effect of caprine herpesvirus 1 (CpHV-1) on mesothelioma cell viability and clonogenicpotential. (**A**) Dose-response curves reporting the effects of five different MOI (multiplicity of infection) of CpHV-1 on cell viability, evaluated through MTS assay at 24, 48, 72, 96, and 120 h post-infection. This assay was performed in three mesothelioma cell lines (NCI-H28, MSTO-211H, and NCI-H2052) and immortalized mesothelial cells (MET-5A). The results are reported as the means ± standard deviation of two independent experiments, each conducted in triplicate, and expressed as percentages of cell viability (calculated with respect to the untreated control cells). (**B**) Long-term CpHV-1 effects were assessed by clonogenic assay. Colonies were stained with crystal violet 10 days after a 72 h treatment with CpHV-1 (5 MOI). Representative plates, out of two (MSTO-211H) or three (NCI-H28, NCI-H2052) independent experiments are shown.

We first assessed, through an MTS assay, the effect of CpHV-1 on MM (NCI-H28, MSTO-211H, NCI-H2052) and on immortalized human normal mesothelial (MET-5A) cell viability at 24, 48, 72, 96, and 120 h after treatment (Figure 1A). The infection showed a time- and dose-dependent cytotoxic effect in all the MM cell lines without meaningfully affecting the normal mesothelial cells. Interestingly, CpHV-1 also induced a cytotoxic effect in the most aggressive sarcomatoid NCI-H2052 cells, which showed resistance to other OVs in previous studies [22,39]. In particular, we observed that, 72 h post-infection (p.i.), a multiplicity of infection (MOI) value of 5 reduced cell viability by approximately 50% in all the MM cell lines.

To verify whether CpHV-1 exerted long-term cell growth inhibition, we performed clonogenic assays upon infection with 5 MOI of CpHV-1 and found that the OV dramatically reduced colony formation in all the MM cell lines. (Figure 1B). The MET-5A did not form clones at the low cell density required for this assay and, therefore, the long-term effect of CpHV-1 was not assessed in this cell line.

### 3.2. Detection of CpHV-1 DNA in MM Cells and Not in Normal Mesothelial Cells

To rule out a possible CpHV-1 replication in normal cells, we infected the non-cancerous MET-5A cells with 5 MOI of CpHV-1. We also treated the MM cells (MSTO-211H) with the same virus dose for comparison. We extracted forty-eight h p.i. and amplified them by real-time PCR viral DNA from both supernatants and adherent cells to estimate the amount of extracellular and intracellular viral DNA, respectively. We quantified this DNA by interpolation from a standard curve constructed with amplified CpHV-1 DNA from serial dilutions of known concentration. Our data confirmed the presence of the CpHV-1 DNA in tumor cells, especially intracellularly, whereas for MET-5A cells, only extracellular viral DNA was detected (Supplementary Figure S1A).

We also infected MET-5A and MSTO-211H with different doses of CpHV-1, 5 MOI and 10 MOI, respectively, and extracted viral DNA after 48 h and 72 h p.i., which was then analyzed by real-time PCR. Our results demonstrated that the ct values (indicative of the amount of viral DNA) did not change in the MET-5A, whereas they were reduced, both in a dose- and a time-dependent manner, in the MM MSTO-211H cells (Supplementary Figure S1B).

Viral production in mesothelial and MM cell lines was tested by harvesting cells and supernatants upon infection with 5 MOI of CpHV-1 for 24 h, 48 h, 72 h, 96 h, and 120 h. Cells and supernatants from each time point were then assayed in the MDBK cells and viral production was reported as Median Tissue Culture Infectious Dose (TCID50). Viral production was observed in all the MM cancer cell lines, whereas no viral production was observed in the normal immortalized cells, MET5A (Supplementary Figure S1B).

### 3.3. CpHV-1 Induces Apoptosis in MM Cells

**Figure 2:**
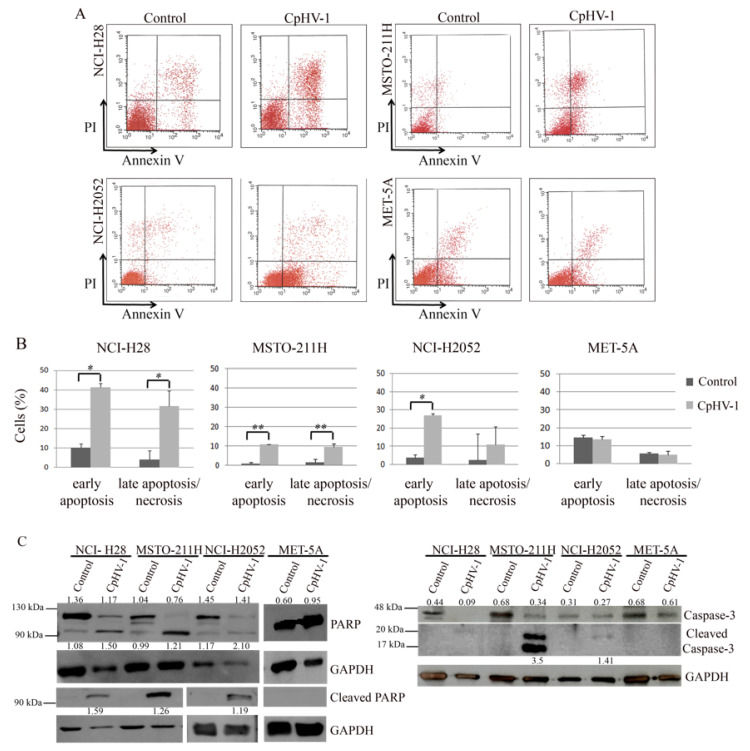
Apoptosis induction in mesothelioma cells infected with caprine herpesvirus 1 (CpHV-1). (**A**) FACS analysis to investigate apoptosis by cell staining with Annexin V-FITC and propidium iodide (PI) in NCI-H28, MSTO-211H, NCI-H2052, and MET-5A cells 72 h post-infection with 5 MOI (multiplicity of infection) of CpHV-1. A representative plot, based on at least two independent experiments, showing early apoptosis (annexin V positivity and PI negativity) and late apoptosis/necrosis (positivity to both annexin V and PI), is reported. (**B**) Histograms reporting the means with standard deviations of at least two independent Annexin V-FITC and PI experiments. Statistically significant differences were evaluated by one-way repeated measures ANOVA with Bonferroni post-test and indicated as follows: * *p* < 0.05; ** *p* < 0.01. (**C**) Western blot analysis of poly (ADP-ribose) polymerase (PARP) and Caspase-3 in NCI-H28, MSTO-211H, NCI-H2052, and MET-5A treated, as reported above. The anti-PARP and anti-caspase-3 antibodies detect both the full-length proteins and the active cleaved form. For caspase-3, the full-length protein and the cleaved form are shown separately at different exposure times (the full-length protein bands are shown at a shorter exposure time because they become overexposed at the time necessary for cleaved caspase-3 bands to appear). An antibody specifically recognizing cleaved PARP was also used. An anti-GAPDH antibody was used for loading control. Band densitometry values indicate the levels of both full-length and cleaved forms of PARP and caspase-3 normalized to GAPDH levels.

To characterize the mechanism underlying CpHV-1’s effects on MM cell viability, we assessed apoptosis induction by CpHV-1 in all the cell lines. We first analyzed, through FACS, double staining with annexin V–FITC and propidium iodide (PI), to detect early apoptosis and late apoptosis/necrosis. Seventy-two hours after infection at 5 MOI, we observed that CpHV-1 induced apoptosis in all the MM cell lines, without affecting the viability of the non-tumor MET-5A cells (Figure 2A,B). Next, to further confirm this finding, we evaluated the activation of the apoptotic markers, poly(ADP-ribose) polymerase (PARP) and caspase-3. As expected, CpHV-1 infection induced a reduction in the full-length proteins and/or an increase in their active cleaved forms in MM cells and not in MET-5A (Figure 2C).

### 3.4. CpHV-1 Perturbs MM Cell Cycle Progression

**Figure 3:**
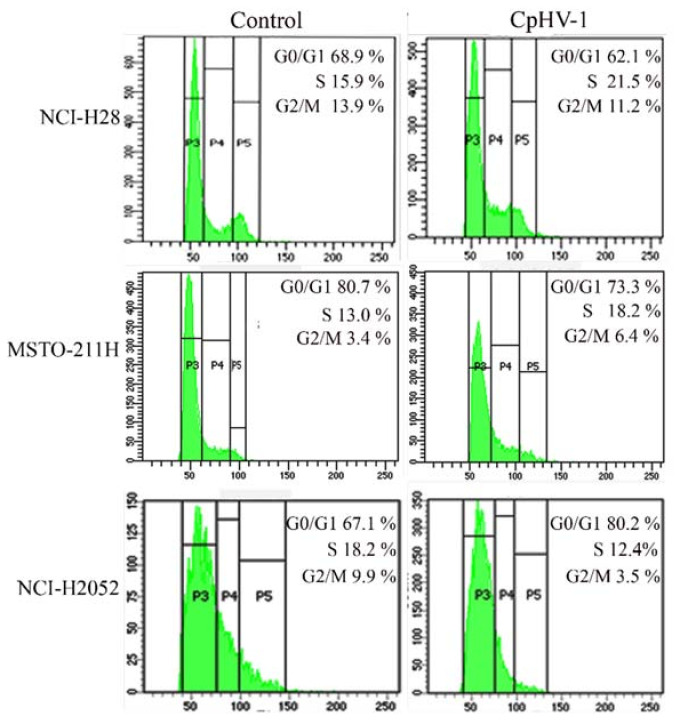
Effect of caprine herpesvirus 1 (CpHV-1) on mesothelioma cell cycle progression. A representative cell cycle profile, based on two independent FACS analyses performed on each MM cell line (NCI-H28, MSTO-211H, and NCI-H2052) upon infection with CpHV-1 (5 MOI, multiplicity of infection), is shown.

Viruses utilize diverse strategies to subvert host cellular response [40,41,42]. They can interfere with the cell cycle, causing a blockage in G0-G1, to induce changes in host cell metabolism that play a crucial role in the viral life cycle [41], or to induce quiescent cells to enter into S phase, creating an environment more advantageous to their replication [40]. Both different mechanisms could depend on the cell cycle phase of the cellular model used [40]. We assessed the effects of the CpHV-1 infection on MM cell cycle progression by evaluating cellular DNA content through FACS analysis 72 h p.i. We found that CpHV-1 affected MM cell cycle phase distribution, although with differences among cell lines. In particular, we observed an increase in the S-phase cell population in the NCI-H28 and MSTO-211 cell lines and an accumulation of NCI-H2052 cells in the G0/G1 phase (Figure 3). Our results are consistent with previous studies showing that HSV-1 is able to alter host cell cycle progression by affecting different phases [40,41,42].

### 3.5. CpHV-1 Synergizes with Cisplatin in Suppressing MM Cell Viability

**Figure 4:**
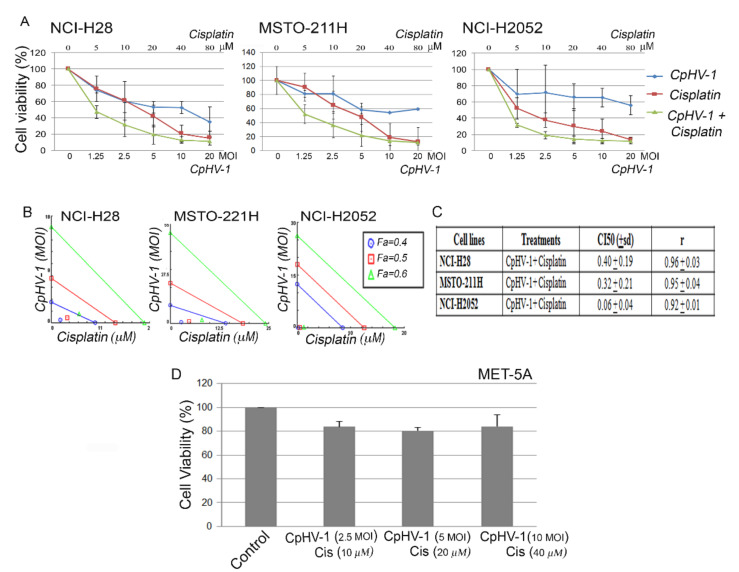
Synergistic effect of caprine herpesvirus 1 (CpHV-1) and cisplatin in mesothelioma cell lines. (**A**) Dose-response curves of CpHV-1 alone, cisplatin alone, and both agents in combination in NCI-H28, MSTO-211H, and NCI-H2052 mesothelioma cell lines 72 h after treatment. Results represent the means ± standard deviation of two independent experiments, each conducted in triplicate, and are expressed as percentages of cell viability calculated with respect to control cells treated with DMSO alone. (**B**) Isobologram analysis to assess synergism between CpHV-1 and cisplatin. Isobolograms are derived from the mean values of the dose–response experiments reported in A, through the Compusyn software at fixed effect levels (Fa, fraction affected) of 40%, 50%, and 60%. The points below the lines indicate synergism. (**C**) Table reporting the means ± standard deviations of combination index (CI) and the r values of the CpHV-1 and cisplatin combination at 50% of cell killing (CI50) following 72 h of treatment, calculated using Compusyn software for each of the two independent experiments. CI values < 1 indicate synergism. (**D**) Histogram showing that 72 h of treatment with CpHV-1 and cisplatin in combination at the indicated doses had no significant toxic effect on the normal mesothelial cells MET-5A, as assessed through MTS and evaluated by one-way repeated measures ANOVA with Bonferroni post-test. Results are reported as means ± standard deviations of two independent experiments and expressed as percentages of cell viability relative to control cells treated with DMSO alone.

We also examined, by MTS, the possible synergistic effects of CpHV-1 in combination with cisplatin, which is the first-line treatment against MM. To this end, we treated the three MM cell lines for 72 h with the two agents, both alone and in combination, at five different concentrations, in a constant ratio. In particular, the agents were added in twofold serial dilutions above and below 5 MOI of the virus and the cisplatin half-maximal inhibitory concentration (IC50) values (as determined in our previously published data [43]) (Figure 4A). The cell viability data were evaluated by isobologram analysis, which showed a strong synergism between CpHV-1 and cisplatin (Figure 4B). Indeed, analysis through the Chou–Talalay method [44] revealed combination index (CI) values < 1 for all cell lines (Figure 4C).

To exclude the possible cytotoxic effects of the combination treatment on non-neoplastic cells, we treated the normal mesothelial cells MET-5A with three different CpHV-1-cisplatin combination doses, which corresponded to the two-agent concentrations, leading to more than 50% reduction in MM cell viability, and observed no significant toxic effect 72 h after treatment (Figure 4D). No significant effects on cell viability were observed in MET-5A with the single agents (2.5, 5 and 10 MOI of CpHV-1 and 10 µM, 20 µM and 40 µM cisplatin), (data not shown).

## 4. Discussion

MM is a universally fatal disease, for which no therapy has proven to be effective [45]. The standard therapeutic approach is mainly based on chemotherapy regimens, which offer limited survival benefit [12]. Despite the advances in the understanding of the molecular mechanisms underlying MM development and although several promising therapeutic strategies, based on targeted and biological agents, have been attempted so far [7,9,10,11,12], patients’ median OS remains approximately 1 year from diagnosis [4,15].

An appealing treatment for this tumor is oncolytic virotherapy, which has acquired an important role in cancer therapy, proving efficacy and safety in many clinical studies [16,17,18,19]. MM is considered especially amenable to treatment with OVs, and, indeed, many studies evaluated the possible use of both replication-competent and incompetent viruses against this disease [21]. We recently demonstrated that the oncolytic adenovirus *dl922-947* exertedantitumor effects in MM cells, both as a single agent and in combinatorial treatments [22,23].

Besides adenovirus, one of the most studied viruses for cancer therapy is oHSV-1 [25]. Indeed, an engineered oHSV-1 (T-VEC) was the first OV to be approved for the treatment of advanced melanoma [25,27]. Moreover, non-human OVs have been extensively studied both in preclinical models and in clinical trials, showing encouraging results [31,32,33,35].

In the present study, we tested the effects of a non-human wild-type OV belonging to the subfamily of the alpha herpesviruses, CpHV-1 [46], on a panel of MM cell lines and normal mesothelial cells. We observed that CpHV-1 reduced the viability and clonogenic potential of all MM cell lines, including the most aggressive sarcomatoid NCI-H2052 cells, without exhibiting cytotoxic effects on normal cells.

To rule out a possible CpHV-1 replication in normal mesothelial cells, we treated MET-5A cells and also MM cells, for comparison, with this OV and extracted and amplified viral DNA from both supernatants and adherent cells to quantify extracellular and intracellular viral DNA, respectively. CpHV-1 DNA was detected only extracellularly in the mesothelial MET-5A cells, whereas it was present both intracellularly and extracellularly in the MM cells.

These findings are consistent with the tumor selectivity of several OVs, which exploit the molecular defects of tumor cells [47]. For instance, HSV uses CD46, a membrane cofactor protein involved in cell fusion, as a receptor to enter cells [48], and this factor is overexpressed in tumor cells, including MM cells [49]. Interestingly, it has been demonstrated that the susceptibility of MM cell lines to attenuated oncolytic measles virus was related to cell surface levels of CD46. Indeed, measles OV preferentially infected MM cells compared with normal mesothelial MET-5A cells, in line with the significantly lower CD46 levels in these normal cells [49]. The glycoprotein H (gH) is the known viral component responsible for binding to CD46 and viral entry in Human herpesvirus 6 (HHV-6) [50] and BoHV-1 [51]. Considering the homology between BoHV-1 and CpHV-1 [34], we also hypothesize a role for these molecules in determining CpHV-1 differential entry in mesothelial/mesothelioma cells. It is not possible to exclude that CpHV-1 enters in normal mesothelial MET-5A cells; however, the functional antiviral pathways or the active cell cycle checkpoint prevents viral replication. We plan to address these points with future experiments.

Cellular changes induced by viral infection are often strikingly similar to those acquired during carcinogenesis [52]. HSV-1, for instance, induces various functional changes that block host defense against infection, such as bypassing the RNA-dependent protein kinase (PKR) pathway [24]. PKR is encoded by an IFN-stimulated antiviral gene (ISG), which is generally upregulated in normal cells after viral infection to inhibit viral genome replication [53]. A previous study demonstrated that MM cells exhibited heterogeneous responsiveness to attenuated oncolytic vesicular stomatitis virus (VSV) based on their ISG expression levels [54]. This study, in particular, showed that VSV infection did not cause cytotoxicity in any normal mesothelial cell lines, consistent with their higher expression of ISGs, including PKR, compared with the responsive MM cell lines [54].

Overall, the studies mentioned above describe some mechanisms underlying the lower susceptibility of normal mesothelial cells to OV infection compared with MM cells. Defining the mechanisms leading to the selective CpHV-1 action on MM cells will be the subject of our future research.

Considering that HSV-1 infection has been previously reported to alter the host cell cycle to create a favorable environment for virus replication [40,41,42], we investigated the effects of CpHV-1 on MM cell cycle progression. Our data showed that CpHV-1 affected the MM cell cycle phase distribution by increasing the S-phase cell population in NCI-H28 and MSTO-211 cell lines and inducing an accumulation of NCI-H2052 cells in the G0/G1 phase. These data are in line with the HSV-1’s ability to affect different cell cycle phases [40,41,42], likely depending on diverse cell contexts. In particular, the HSV-1-induced increase in the S-phase cell population has been suggested to facilitate virus replication [41], whereas the G1 cell cycle arrest might favor the activation of viral gene expression [32].

We also observed that CpHV-1 induced apoptosis in all the MM cell lines, as indicated by positivity to annexin V and activation of caspase-3 and PARP. As expected, MET-5A cells did not show any of these hallmarks of apoptosis. These results are consistent with our previous published data, demonstrating that CpHV-1 induces apoptosis in several human cancer cell lines [35].

Importantly, we previously observed that the oncolytic adenovirus *dl922-947* did not affect the viability of NCI-H2052 cells [22], which is consistent with their resistance to the infection with Ad5 derived OVs [39]. Conversely, CpHV-1 infection was also effective against this cell line, suggesting that this virus could represent an alternative viral treatment against MM.

Although OVs are potentially powerful therapeutic agents against cancer, they frequently prove to be more effective in combinatorial treatments and are often tested in combination with standard chemotherapeutic agents [19,22,55]. In the present study, we observed that CpHV-1 strongly synergized with cisplatin, and this agent combination exerted no toxic effect on mesothelial cells. Thus, our data suggest the possible use of CpHV-1 to sensitize MM cells to cisplatin treatment. It has previously been shown that the cellular stress response induced by cisplatin in MM cell lines can potentiate the replication and cytotoxicity of NV1066, a replication-competent oncolytic HSV-1, attenuated by a deletion in the gene gamma (1) 34.5 [56]. In particular, this study demonstrated that cisplatin-induced GADD34 (growth arrest and DNA damage-inducible protein) expression enhanced the cytotoxicity of NV1066 in MM cells [54]. These data are consistent with our results and further support the combination of cisplatin and oncolytic herpesviruses as an effective strategy against MM. Here, we used a non-human, non-engineered OV, which could offer some advantages over human attenuated OVs because viral attenuation could reduce therapeutic success [28].

Further studies are required to evaluate CpHV-1’s ability to trigger an immune response against MM cells and modulate the tumor microenvironment toward an antitumoral phenotype.

In conclusion, although future research is needed to elucidate the CpHV-1 mechanisms of action both in MM cell lines and animal models, we observed that CpHV-1 induced apoptosis and strongly synergized with cisplatin, without affecting non-neoplastic mesothelial cells, thus suggesting that this selective therapeutic approach is a promising new strategy for the treatment of MM.

## Data Availability

Not applicable.

## Supplementary Materials

The following are available online at https://www.mdpi.com/article/10.3390/v13122458/s1, Figure S1: (A) Viral DNA quantification, (B) Viral production, Figure S2: Effect of caprine herpesvirus 1 (CpHV-1) on mesothelioma cell viability.

## Abbreviations

CI	Combination index
CpHV-1	Caprine Herpesvirus-1
Fa	Fraction affected
gH	glycoprotein H
HSV-1	Herpes Simplex Virus type 1
IC50	Half maximal inhibitory concentration
ISGs	IFN-stimulated antiviral genes
MM	Malignant Mesothelioma
MOI	Multiplicity of infection
oHSV-1	Oncolytic Herpes Simplex Virus type 1
OS	Overall survival
OV	Oncolytic Virus
PARP	Poly (ADP-ribose) polymerase
PI	Propidium iodide
p.i.	Post-Infection
PKR	RNA-dependent protein kinase
PTCID50	Median Tissue Culture Infectious Dose
T-VEC	Talimogenelaherparepvec
VSV	Vesicular stomatitis virus
