# Supplementary material for: The Oncolytic Caprine Herpesvirus 1 (CpHV-1) Induces Apoptosis and Synergizes with Cisplatin in Mesothelioma Cell Lines: A New Potential Virotherapy Approach
Source: Viruses. 2021 Dec 8;13(12):2458. doi: 10.3390/v13122458 (PMC8703924; doi:10.3390/v13122458)
Supplement: Supplementary file 1 [file viruses-13-02458-s001.zip › Supplementary Figure S1.pdf]

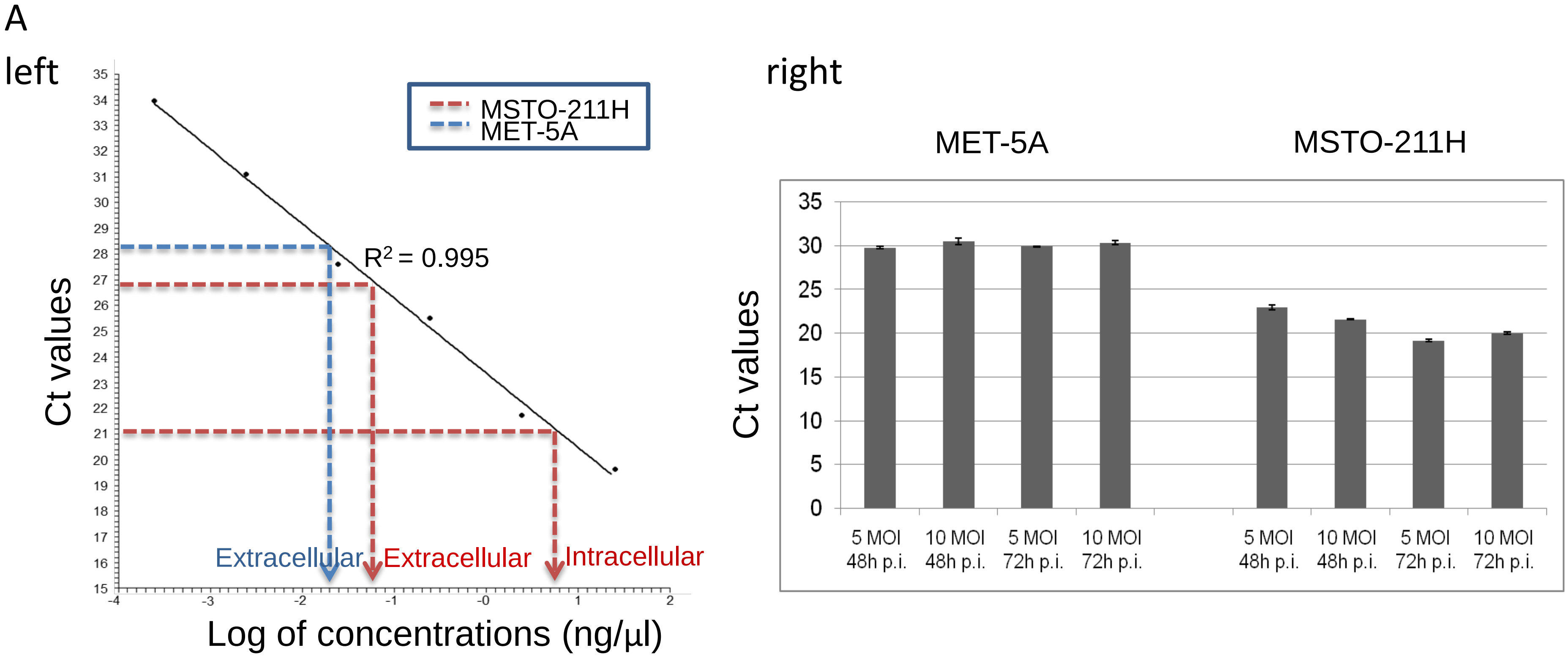

Supplementary Figure S1A. Left: Viral DNA quantification. Normal immortalized mesothelial cells (MET-5A) and mesothelioma cells (MSTO-211H) were infected with 5 MOI (multiplicity of infection) of caprine herpesvirus 1 (CpHV-1). Forty-eight h post-infection, viral DNA was amplified by real-time PCR from both supernatants and adherent cells to estimate, respectively, the amount of extracellular and intracellular viral DNA. DNA was quantified by interpolation from a standard curve constructed with amplified CpHV-1 DNA from serial dilutions of known concentration. CpHV-1 DNA was detected in mesothelioma cells (especially intracellularly), whereas for MET-5A cells, only extracellular viral DNA was detected. Right: MET-5A and MSTO-211H cells were also infected with different doses of CpHV-1, 5 MOI and 10 MOI respectively. Viral DNA was extracted 48h and 72h post infection and amplified by real-time PCR. The graph shows the media ± standard deviation of ct values of two independent experiments

B

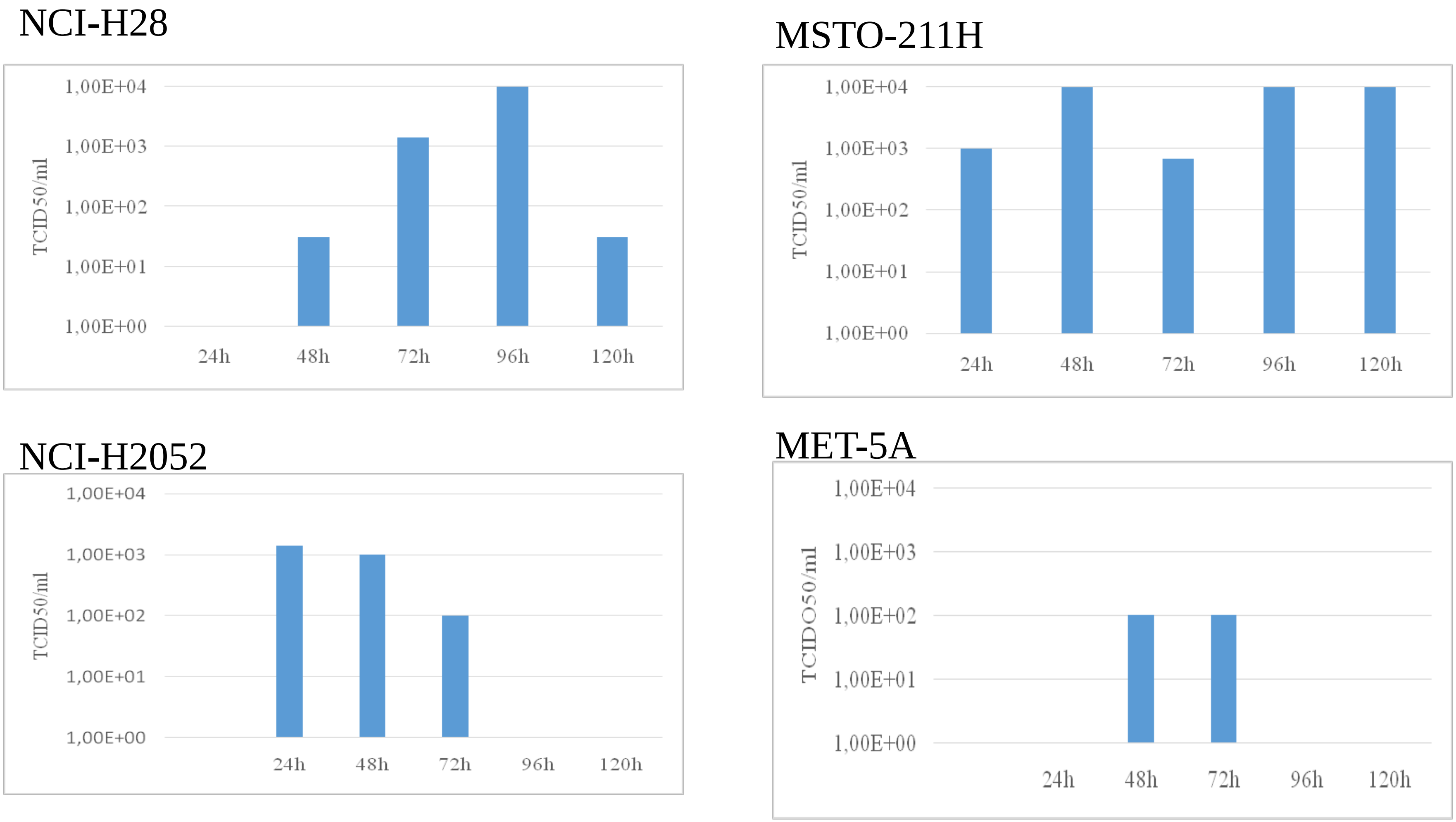

Supplementary Figure S1B. Viral production. Mesothelioma cells (NCI-H28, MSTO-211Hand NCI-H2052) and normal immortalized mesothelial cells (MET-5A) were infected with 5 MOI (multiplicity of infection) of caprine herpesvirus 1 (CpHV-1) for 24h, 48h, 72h, 96h and 120h. Cells and supernatants from each timepoint after three cycles of freezing and thawing, were then assayed in MDBK cells and viral production was reported as Median Tissue Culture Infectious Dose (TCID50).

The graphs show a time course of virus yield, in supernatants and cell-associated virus and the viral production was observed in all MM cancer cell line, while no efficient viral replication was observed in normal immortalized cells, MET5A. The Cytopathic effect (CPE) was scored and calculated using the Reed and Muench method [38]
