# Supplementary material for: The Oncolytic Caprine Herpesvirus 1 (CpHV-1) Induces Apoptosis and Synergizes with Cisplatin in Mesothelioma Cell Lines: A New Potential Virotherapy Approach
Source: Viruses. 2021 Dec 8;13(12):2458. doi: 10.3390/v13122458 (PMC8703924; doi:10.3390/v13122458)
Supplement: Supplementary file 1 [file viruses-13-02458-s001.zip › Supplementary Figure S2.pdf]

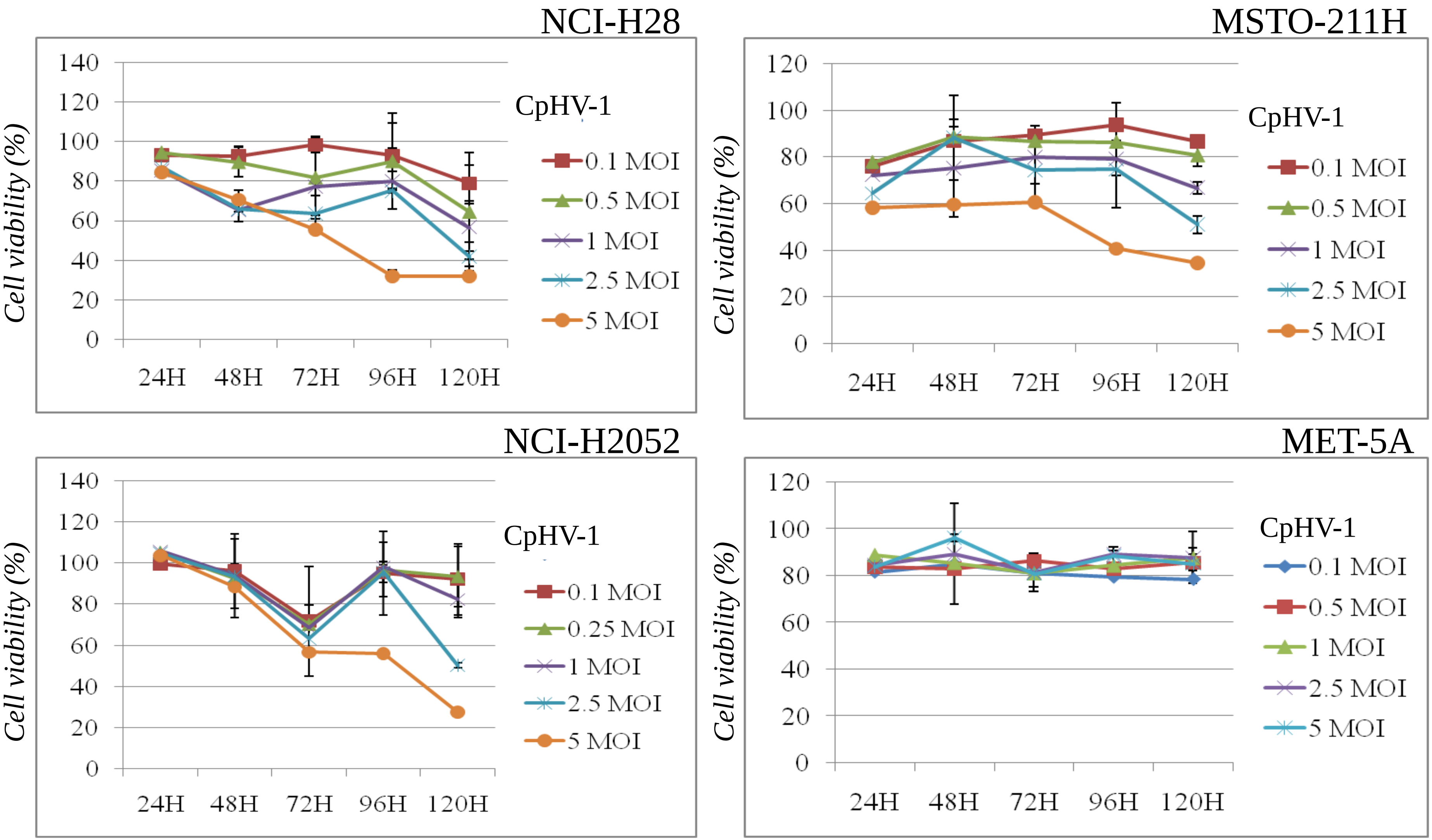

Supplementary Figure S2: Effect of caprine herpesvirus 1 (CpHV-1) on mesothelioma cell viability. Dose–response curves reporting the effects at 24, 48, 72, 96, and 120 h post-infection of five different MOI (multiplicity of infection) of CpHV-1 on cell viability, evaluated through MTS assay. This assay was performed in three mesothelioma cell lines (NCI-H28, MSTO-211H, and NCI-H2052) and normal immortalized mesothelial cells (MET-5A). The results are reported as the means ± standard deviation of two independent experiments, each conducted in triplicate, and expressed as percentages of cell viability (calculated with respect to the untreated control cells).
